# A new era for mind studies: training investigators in both scientific and contemplative methods of inquiry

**DOI:** 10.3389/fnhum.2013.00741

**Published:** 2013-11-05

**Authors:** Gaëlle Desbordes, Lobsang T. Negi

**Affiliations:** ^1^Athinoula A. Martinos Center for Biomedical Imaging, Massachusetts General HospitalBoston, MA, USA; ^2^Center for Computational Neuroscience and Neural Technology, Boston UniversityBoston, MA, USA; ^3^Department of Religion, Emory UniversityAtlanta, GA, USA

**Keywords:** contemplative science, subjective experience, neurophenomenology, consciousness, meditation

The study of the human mind has evolved over the course of many centuries. While modern neuroscience relies on objective, quantitative methods for measuring how mental events manifest as brain activity, ancient contemplative traditions have used first-person introspective practices to gain a greater understanding of the mind. It is now possible to combine these different approaches, hopefully in a mutually enriching, synergistic way. As proposed by the late Francisco J. Varela, studying the conscious mind could greatly benefit from bringing together the “first-person” perspective of a well-trained experimental subject with the “third-person” perspective of an outside observer (i.e., a scientist measuring brain activity). Varela called this approach neurophenomenology, referring to the combination of neural measurements with the style of inquiry of classic phenomenology (Varela, 1996). In neurophenomenology experiments, the subject is actively involved in describing his moment-by-moment conscious experience and is sometimes asked to generate specific mind states, while the experimenter is guided by these first-person data in the analysis and interpretation of physiological data (Lutz and Thompson, 2003). This methodology can be used to better account for seemingly random fluctuations in brain activity, which are usually discarded as “noise” but may reveal key insights into ongoing variations in the subject's inner experience (Lutz et al., 2002; Lachaux, 2011).

Recent advances in neuroscience may enable, for the first time, direct testing with rigorous scientific methods of claims made in past centuries through contemplative practices about how the mind works. Technological tools for measuring brain activity are becoming more precise and more sophisticated every day. However, high quality first-person data remain difficult to obtain. Introspective methods have been criticized on the account that most mental processes are usually not consciously accessible. For example, we are usually unaware of our own decision-making processes (Nisbett and Wilson, 1977), and sensory stimuli can be perceived even in the absence of conscious awareness (Merikle et al., 2001). Nevertheless, rigorous introspection methods such as the elicitation interview developed by Petitmengin (2006) enable participants to provide—and validate—detailed first-person accounts of their mental experience (Bitbol and Petitmengin, 2013). These new developments in introspective methods have challenged the notion that mental processes that are usually unconscious are irremediably inaccessible to conscious awareness, and suggest that some of them may be revealed when using appropriate methods (Bockelman et al., 2013; Petitmengin and Lachaux, 2013; Petitmengin et al., 2013).

Expert contemplative practitioners are particularly well suited to introspective methods, as many of their practices require them to observe and describe their experience on a moment-by-moment basis in exquisite detail—thereby providing extremely valuable first-person data in the context of a neurophenomenological experiment. Expert meditation practitioners have acted as experimental subjects in a number of neuroscientific studies to date, although only few studies have included first-person input from these subjects as an essential aspect of their methods (e.g., Carter et al., 2005; Dor-Ziderman et al., 2013; Garrison et al., 2013a,b).

In addition to being valuable study participants, expert meditators and contemplative scholars could contribute to scientific studies in other ways, as proposed by Varela (Varela et al., 1992; Lutz and Thompson, 2003). The traditional curriculum received by these experts typically includes not only training in contemplative practices but also extensive knowledge of the conceptual and cultural frameworks in which they have developed over the centuries. In this regard, the scientific study of the mind would benefit immensely from including contemplatives as full-fledged co-investigators. Importantly, such collaborations require establishing sufficient common ground between the perspectives of contemplatives and scientists, which can be difficult for individuals exclusively trained in one or the other discipline, as became evident in the first attempted interactions between both sides. The Mind and Life conferences have opened the way by facilitating exchanges between scientists and His Holiness the Dalai Lama on a number of scientific topics over the years, including topics relevant to the study of the mind, such as consciousness (Houshmand et al., 1999; Hayward and Varela, 2001; His Holiness the Dalai Lama, and Varela, 2002), emotions (Goleman, 1997, 2003), and neuroplasticity (Begley, 2007), and some of these meetings have inspired new scientific studies. More broadly, these ground-breaking initiatives have coalesced into the creation of a new, multi-disciplinary field of research called contemplative science.

However, to date, individuals with expertise in both contemplative practice and modern science have been so rare as to be exceptional. Perhaps the most well-known of them is Matthieu Ricard, who holds a doctorate degree in molecular biology and was fully trained as a scientist up to the postdoctoral level, and then fully trained as a monk in the Tibetan Buddhist tradition. “Matthieu-la” (as he is affectionately known among his peers at Shechen Monastery) has been a close collaborator with a number of scientists, including Richie Davidson, Antoine Lutz, Paul Ekman, Tania Singer, and others (Lutz et al., 2004; Ekman et al., 2005; Levenson et al., 2012; Klimecki et al., 2013b).

The field of contemplative science would benefit immensely from increasing the pool of individuals trained in both contemplative practice and modern science. Targeted education efforts are needed to bridge this gap, and many of them are already ongoing. On the science side, rigorous programs for contemplative training are now offered to college students, scientists, clinicians, and other professionals, such as those provided by the Center for Mindfulness in Medicine, Health Care, and Society at the University of Massachusetts Medical School, the Contemplative Studies Initiative at Brown University, the Center for Compassion and Altruism in Research and Education at Stanford University through the Compassion Cultivation Training program, and the Emory-Tibet Partnership at Emory University through programs such as Cognitively-Based Compassion Training and the Tibetan Mind/Body Sciences Program.

On the contemplative side, many efforts have been made in particular in the Tibetan Buddhist community to provide scientific training to monastics. Topical science workshops have been offered to monastics over the past decade by science volunteers as part of various programs such as Science for Monks, Science Meets Dharma, and others. A more advanced training has been provided for a smaller number of monks and nuns to deepen their scientific knowledge and promote scientific awareness in their respective monasteries through teaching and science exhibits (Phayul, 2010). On a broader scale, the Robert A. Paul Emory-Tibet Science Initiative (or ETSI) is a historic endeavor to design and implement a multi-disciplinary science education curriculum specifically aimed for Tibetan monastics, as part of a large-scale effort envisioned by His Holiness the Dalai Lama (Yee, 2009). This initiative has involved dozens of faculty from the fields of neuroscience, biology, cosmology, and philosophy of science—as well as several science-trained Tibetan translators. To ensure the long-term sustainability of the program by training indigenous Tibetan monastic science teachers, the ETSI has also established the Tenzin Gyatso Science Scholars program, which has enabled six Tibetan monks to spend three academic years studying science at Emory University, with another cohort starting in the Fall of 2013 (Severson, 2013).

Language and terminology translation is an important component of the ETSI, with the ambitious long-term goal to create a new scientific vocabulary in the Tibetan language, in collaboration with the Library for Tibetan Works and Archives in Dharamsala, India. It should be noted that the problem of translation exists both ways. Not only does the Tibetan language lack words to describe modern scientific concepts, but the English language also lacks precise words to describe nuanced aspects of mind and consciousness as found in Tibetan, Sanskrit, Pali, Chinese, and other languages from contemplative traditions. For example, even the broad term “mind” in English does not have an exact equivalent in Tibetan and may be translated as *sems, blo, shepa*, or other words depending on context. The development of a consensual lexicon to represent these precise concepts in the English language is therefore also much needed to further the scientific study of the mind.

The ETSI has led to the creation of a 5 year science education curriculum, which has already been offered to two groups of carefully selected monks and nuns already well advanced in their Buddhist studies, totaling over ninety students. Now entering its second phase, the ETSI will then implement this 5 year curriculum on a larger scale to several major monasteries within Tibetan Buddhism. This new endeavor follows a recent, historic decision to include science education (and exams) to the official monastic curriculum—a decision that is particularly significant given that the training in these monasteries had remained unchanged for the past 500 years (Phayul, 2012). This move toward modern science, considered improbable several years ago, is still controversial for some but has certainly generated a lot of interest in the monastic community, as attested by the enthusiasm at the recent Mind and Life XXVI meeting at the Drepung monastery in Mundgod, India, the first of its kind to be taking place in a Tibetan monastery with thousands of monastics in attendance (De Rothschild, 2013).

It is our hope that some of these contemplative experts trained in modern sciences, together with a growing number of scientists trained in contemplative practices, will be able to contribute their unique perspectives and qualifications toward future collaborative scientific studies of the mind. Some promising avenues for these collaborations include the study of cognitive processes such as attention, perception, and decision-making, but also emotional and social processes. In addition, we believe that collaborative endeavors between neuroscientists and contemplatives within the neurophenomenological framework may provide new perspectives toward the scientific study of morality and ethics, and their proposed foundation in altruism and compassion (Goetz et al., 2010; Dalai Lama, 2011; Ozawa-de Silva et al., 2012). Indeed there is a fast-growing, unprecedented interest in the scientific community in investigating compassion and altruism, from the perspective of disciplines as varied as neuroscience, social and behavioral science, political science, and economics (Ricard, 2013; Singer and Bolz, 2013). Seminal studies of the neural underpinnings of compassion indicate that extensive compassion training over the course of a lifetime may alter brain networks associated with emotion, attention, and empathy (Lutz et al., 2004, 2008; Brewer et al., 2011) and that even short-term training in loving-kindness and compassion may affect brain function and promote altruistic behavior (Desbordes et al., 2012; Condon et al., 2013; Klimecki et al., 2013a,b; Mascaro et al., 2013; Weng et al., 2013). The inclusion of first-person accounts of the experience of compassion would be an invaluable addition to its scientific study, as others have noted (Halifax, 2012; Kok, 2013). We look forward to future studies of altruism and compassion that skillfully combine scientific and contemplative methods.
